# The Impact of Environmental Exposures on Cystic Acne: A Narrative Review

**DOI:** 10.3390/biomedicines14102381

**Published:** 2026-10-19

**Authors:** Angela Vaysman, Morgan Ulczak, Carly Achterberg, Lucas Goetz, Luke Atwood, Reed Hildenbrant, Erin Hallman, Caitlyn Shaughnessy, Karen Vo

**Affiliations:** 1 School of Medicine, St. George’s University, True Blue, St. George’s, Grenada; ulczakmorgan@gmail.com (M.U.); latwood@sgu.edu (L.A.); rhildenb@sgu.edu (R.H.); 2 Sanford School of Medicine, University of South Dakota, Vermillion, SD 57069, USA; carly.haring@coyotes.usd.edu (C.A.); lucasgoetz5@gmail.com (L.G.); 3 College of Osteopathic Medicine, Michigan State University, East Lansing, MI 48824, USA; hallmane@msu.edu; 4 College of Osteopathic Medicine, Kansas City University, Kansas City, MO 64106, USA; caitlyn.shaughnessy@kansascity.edu; 5 Desert Regional Medical Center, Palm Springs, CA 92262, USA; karen.vo@tenethealth.com

**Keywords:** cystic acne, acne vulgaris, agricultural exposures, airborne irritants, occupational dermatology, skin barrier, inflammation

## Abstract

Cystic acne is a severe inflammatory dermatological condition traditionally associated with genetic, hormonal, and microbial factors. Although environmental exposures may contribute to acne pathogenesis, the role of airborne agricultural irritants remains underexplored. Agricultural environments contain a complex mixture of organic dusts, pesticides, fertilizers, volatile compounds, and animal-derived particulates that may influence skin barrier integrity and inflammatory responses. This narrative review examines the potential relationship between airborne agricultural exposures and cystic acne by integrating evidence from dermatology, environmental health, and immunology. Particular attention is given to mechanisms through which agricultural irritants may disrupt epidermal barrier proteins, including filaggrin and claudins; promote oxidative stress and lipid peroxidation; alter the cutaneous microbiome; and activate inflammatory signaling pathways, including nuclear factor kappa B (NF-κB) and the NLRP3 inflammasome. These processes may contribute to the release of proinflammatory cytokines, including interleukin (IL)-1β, IL-6, tumor necrosis factor-alpha (TNF-α), and IL-8, potentially amplifying inflammation within the pilosebaceous unit. The review also explores the possibility that chronic inhalation of agricultural aerosols may contribute to systemic immune activation and influence cutaneous inflammatory responses. While environmental and occupational exposures have been associated with inflammatory skin conditions, direct evidence linking airborne agricultural irritants specifically to cystic acne remains limited. This review highlights potential mechanistic connections, identifies gaps in the current literature, and emphasizes the need for further epidemiological and experimental research to clarify these associations. Understanding the relationship between agricultural airborne exposures and acne may inform future preventive strategies and occupational health considerations.

## 1. Introduction

While cystic acne is traditionally associated with genetic, hormonal, and microbial factors, emerging evidence suggests that environmental exposures may also influence its development and severity. Acne is highly prevalent and can impose substantial psychological and social burdens, particularly among adolescents and young adults [1]. Although its pathogenesis is multifactorial, the contribution of exogenous irritants to acne-related inflammation remains incompletely understood [2]. Understanding environmental factors that may exacerbate acne is therefore important for improving disease prevention and occupational s54kin health. Given the overlapping terminology used to describe acne-related outcomes, Table 1 distinguishes cystic acne from related conditions, including nodulocystic acne, severe inflammatory acne, and acneiform eruptions.

Agricultural environments present a complex mixture of potential skin irritants, including organic dusts, microorganisms, fertilizers, pesticides, and animal-derived particulates. Exposure to these agents has been associated with oxidative stress, inflammatory responses, and adverse dermatologic outcomes among agricultural workers [3]. The skin may be exposed through both direct contact and airborne deposition, particularly in occupational settings involving repeated or prolonged exposure. Such exposures may compromise cutaneous barrier integrity and contribute to inflammatory processes relevant to acne pathogenesis. However, the extent to which agricultural airborne irritants specifically contribute to cystic acne remains unclear.

Several biological mechanisms support a potential association between agricultural exposures and acne-related inflammation. First, chemical irritants and particulate matter may disrupt the stratum corneum and impair skin barrier function, potentially increasing susceptibility to further irritation and inflammatory signaling [4]. Second, exposure to agricultural chemicals may promote the generation of reactive oxygen species (ROS), contributing to oxidative stress and cellular injury [5]. Third, environmental exposures may alter the cutaneous microbiome, potentially influencing microbial interactions and inflammatory responses within the skin. Finally, inhaled or dermally absorbed irritants may activate immune pathways and contribute to systemic or local inflammation [6]. Although these mechanisms provide biological plausibility, direct evidence establishing a causal relationship between agricultural airborne exposures and cystic acne remains limited.

**Figure 1:**
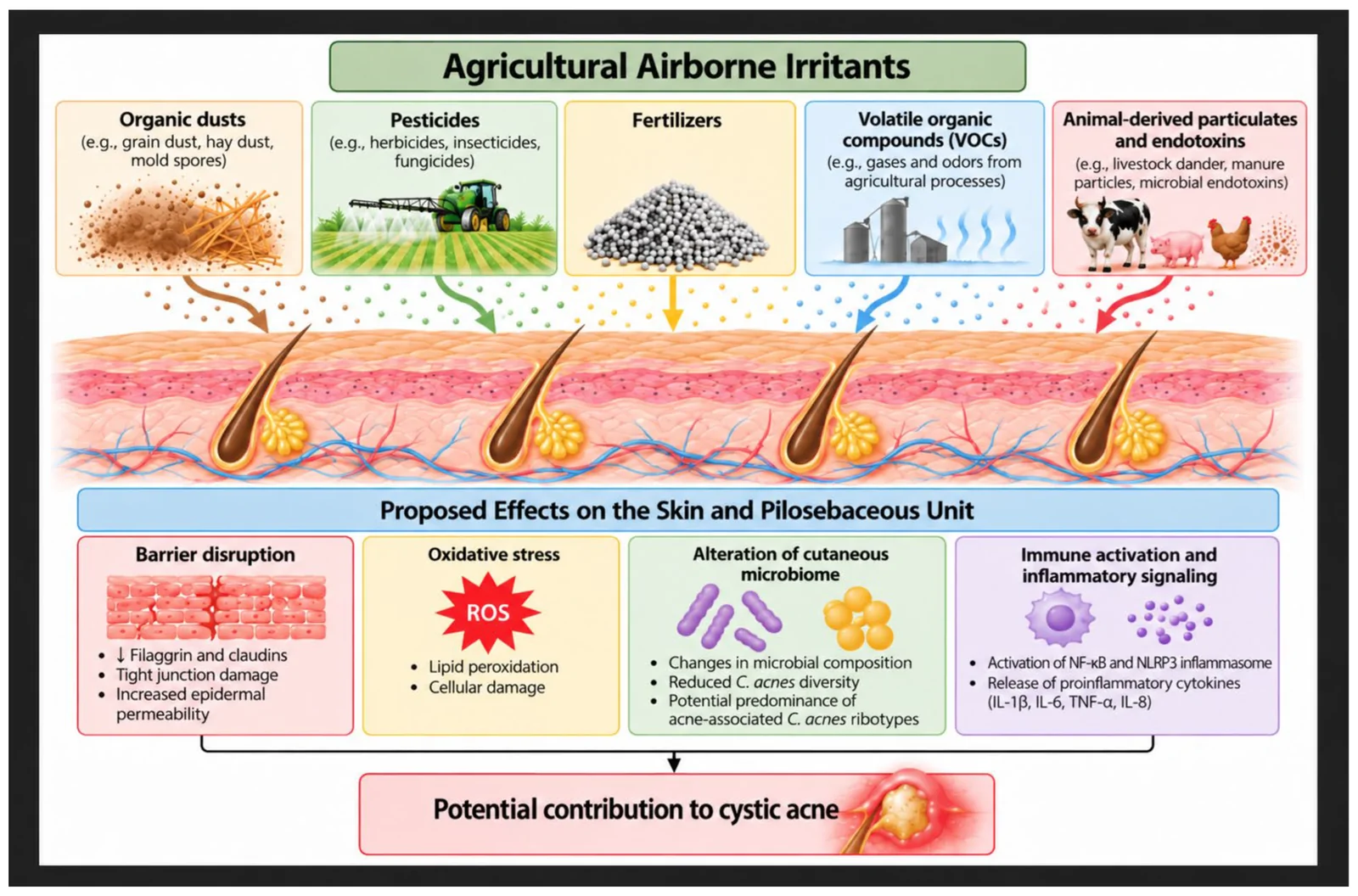
Proposed effects of agricultural airborne irritants on the skin and pilosebaceous unit. Agricultural exposures, including organic dusts, pesticides, fertilizers, volatile organic compounds (VOCs), animal-derived particulates, and endotoxins, may contribute to epidermal barrier disruption, oxidative stress, alterations in the cutaneous microbiome, and inflammatory signaling, potentially contributing to cystic acne. The figure was created with the assistance of OpenAI ChatGPT for AI-generated graphical elements and was reviewed and edited by the authors for accuracy.

This narrative review examines the current evidence linking agricultural airborne irritants with acne-related dermatologic outcomes. By integrating findings from dermatology, environmental health, immunology, and related fields, we aim to evaluate the potential roles of organic dusts, pesticides, fertilizers, and other agricultural exposures in skin barrier disruption, oxidative stress, microbiome alterations, and inflammatory signaling. The proposed effects of agricultural airborne irritants on the skin and pilosebaceous unit are summarized in Figure 1. Overall, the available evidence suggests that agricultural airborne irritants may contribute to acne-related inflammation through convergent biological pathways, while highlighting the need for further epidemiological and mechanistic research to clarify their role in the development and exacerbation of cystic acne.

**Table 1:** Comparison of cystic acne, nodulocystic acne, severe inflammatory acne, and acneiform eruptions.

Condition	Definition	Distinguishing Features
Cystic acne	Large pus-filled cysts beneath the skin	Painful, deep-seated lesions; mixed inflammatory lesions present; scarring common [7]
Nodulocystic acne	Coexisting nodules and cysts	Deep, painful nodules and cysts; leads to permanent scarring [8]
Severe inflammatory acne	Numerous papules, pustules, and nodules	Defined by overall inflammation severity, extent of lesion, which may result in tissue destruction [9]
Acneiform eruptions	Papules, pustules, nodules, cysts	Drug or disease-triggered lesions; comedones uncommon [10]

## 2. Agricultural Airborne Irritants

### 2.1. Organic Dusts: Grain Dust, Hay Dust, and Mold Spores

Agricultural dust, including grain, hay, and mold spores, can promote cutaneous inflammation and barrier disruption. Grain dust contains organic plant particles, heavy metals, and polycyclic aromatic hydrocarbons that can penetrate the skin barrier and trigger dermatitis and other inflammatory conditions [11]. Mold spores, particularly Aspergillus fumigatus, can activate Th17 and Th2 pathways, increase inflammatory cytokine production, and impair regulatory T-cell function with chronic exposure [12]. Airborne soil dust can further irritate the skin through mechanical abrasion and prolonged contact with irritants and microbes, worsening inflammation and barrier damage [13]. Collectively, these exposures can compromise skin integrity and promote inflammatory responses associated with occupational skin disease (Table 2).

### 2.2. Microbial Endotoxins and Their Inflammatory Potential

Endotoxins derived from gram-negative bacteria are widely present in agricultural bioaerosols and organic dust and can reach concentrations that pose health risks [14]. Inhalation induces systemic immune responses, including changes in CD4+ and CD8+ T-cell populations, while smaller endotoxin-bearing particles can penetrate more deeply and interact with tissues beyond the respiratory tract. Endotoxins can also activate epithelial and immune cells, contributing to systemic inflammation, skin irritation, allergic responses, and worsening of preexisting dermatoses. Lipopolysaccharides in dust can directly activate skin immune cells, including neutrophils and lymphocytes, producing localized inflammation and elevated fibronectin levels that may damage tissue and interfere with repair [6]. Together, these effects link endotoxin exposure to systemic immune activation and direct skin injury.

### 2.3. Chemical Exposures: Pesticides, Herbicides, and Fertilizers as Airborne Particulates

Pesticides can disrupt cellular homeostasis and activate inflammatory pathways in exposed tissues. Upon epithelial contact, they can activate Toll-like receptors and induce alarmins such as IL-25, IL-33, TSLP, and HMGB1, stimulating cytokine release and neutrophil recruitment [6]. Organophosphates and pyrethroids can further increase IL-6, IL-1β, and TNF-α, promoting tissue damage and immune activation. Glyphosate- and glufosinate-containing herbicides are often combined with surfactants that have demonstrated severe cellular toxicity in vitro, with greater penetration through damaged or aged skin [18]. Dermal exposure has been associated with swelling, bullae, exuding wounds, chemical burns, and necrosis [18]. Pesticide exposure can also cause dermatitis and burns and alter B-lymphocyte counts and biochemical markers of immune dysregulation [15]. Together, these exposures can contribute to acute skin injury and chronic inflammation.

### 2.4. Volatile Organic Compounds (VOCs) in Barns and Storage Facilities

Volatile organic compounds (VOCs) in barns and storage facilities can adversely affect skin health. VOCs such as toluene downregulate filaggrin, a key protein maintaining skin barrier integrity, while controlled exposure studies in patients with atopic dermatitis demonstrate increased transepidermal water loss [20]. VOCs also promote oxidative stress, generating reactive oxygen species that alter lipid composition, oxidize squalene, and reduce protective fatty acids, processes that may exacerbate acne lesion formation [21,22]. Activation of the aryl hydrocarbon receptor further increases inflammatory signaling, while combined exposure with particulate matter elevates pro-inflammatory cytokines that may worsen acne symptoms [21]. Heavy metals such as lead and cadmium, often present alongside VOCs, can suppress antioxidant defenses and amplify oxidative injury [22]. These effects demonstrate how VOC exposure can impair skin barrier function and promote inflammatory and oxidative processes associated with acne.

### 2.5. Animal-Derived Particulates: Dander, Dried Manure Particles, and Feather Fragments & Potential Contribution to Follicular Occlusion and Inflammation

Agricultural workers are exposed to animal-derived and manure-related particulates that can cause inflammatory skin effects. Farmyard manure and cow dung exposure are associated with higher skin disease prevalence, while manure-related irritants such as ammonia can cause irritation, allergic reactions, and chemical burns [19]. Protective measures such as gloves and footwear reduce disease prevalence, highlighting the importance of avoiding direct skin exposure [19]. Airborne animal dander and related particulates carry allergenic proteins that can penetrate the skin barrier and activate dendritic cells and macrophages, promoting pro-inflammatory cytokine and chemokine release [23]. Some particulates can also activate inflammasomes and amplify inflammatory signaling [20]. These exposures can therefore contribute to direct irritation, allergic responses, barrier dysfunction, and persistent inflammation.

## 3. Skin Barrier Function and Irritant Responses

### 3.1. Structure and Role of the Skin Barrier

The skin is the body’s first line of defense against the external environment. It consists of the epidermis, dermis, and subcutaneous fat. The epidermis is the outermost layer and is subdivided from innermost to outermost into the stratum basale, spinosum, granulosum, and corneum. The skin protects the body from external exposures through four interconnected barrier systems: physical, immunologic, microbial, and chemical.

The physical barrier is primarily located within the stratum corneum, which contains corneocytes within a lipid matrix of ceramides, cholesterol, and free fatty acids [31]. This matrix is derived from lamellar bodies secreted by cells of the stratum granulosum [32]. Surrounding the corneocytes, the hydrophobic lipid matrix restricts permeability to certain chemicals, including water [31]. Structural proteins further strengthen the skin barrier. Filaggrin aggregates keratin to form the stratum corneum, limit epidermal water loss, and provide a structural barrier against allergens and toxins [33]. Another important feature of the stratum granulosum is the presence of tight junctions between keratinocytes. These junctions seal intercellular spaces and limit molecular movement through the paracellular pathway [34]. Tight junctions not only provide an additional physical barrier but also interact closely with the immune system through their relationship with Langerhans cells.

The immunologic barrier works closely with the skin’s structural components to recognize barrier disruption and initiate immune responses to pathogens. Langerhans cells are located just below tight junctions and play an important role in innate immunity [34]. Their proximity to tight junctions allows immune surveillance within the skin barrier. When activated, Langerhans cells extend dendrites through tight junctions to capture antigens that penetrate the stratum corneum and present them to resident regulatory and memory T cells [33,34]. Keratinocytes also contribute to immune responses through cytokine signaling. They express pattern recognition receptors that recognize pathogen-associated and damage-associated molecular patterns, activating NF-κB or MAPK pathways and resulting in inflammatory gene transcription, cytokine signaling, and cell-mediated responses [33]. This communication among keratinocytes, Langerhans cells, and T cells ensures that the skin functions as both a barrier and an immune organ.

The chemical barrier protects the skin through several mechanisms. Lipids in the stratum corneum protect against epidermal water loss, ultraviolet radiation, oxidation, and pathogens [33]. The skin’s acidic pH also protects against dysbiosis and supports enzymatic processes involved in barrier regeneration [33]. This weakly acidic pH of 5.6–6.4 is maintained by free fatty acids in the stratum corneum, lactic acid from eccrine glands, and urocanic acid derived from filaggrin degradation [34]. Skin pH also regulates lipid metabolism and bilayer structure within the stratum corneum, which are crucial for maintaining selective barrier permeability [33]. Exposure to alkaline chemicals can disrupt skin pH and these critical functions.

Finally, the microbial barrier is established by antimicrobial peptides and lipids within the epidermis. Key peptides produced by keratinocytes include cathelicidins and β-defensins, which directly disrupt microbial membranes and modulate immune responses [34]. Antimicrobial lipids secreted by eccrine, apocrine, and sebaceous glands contribute to the skin’s low pH, inhibiting microbial growth [34]. These peptides and lipids help establish the skin microbiome, along with factors such as temperature, anatomic location, hormone status, and age [33]. The microbiome is well adapted to the skin and competes with pathogens, supporting the skin’s immune barrier function.

Together, the physical, immunologic, chemical, and microbial components of the skin barrier defend the body from the external environment. Despite this multilayered defense, airborne irritants possess unique properties that can compromise barrier integrity. Understanding how these exposures disrupt the skin barrier is critical to appreciating their role in inflammatory skin disease.

### 3.2. Barrier Disruption by Airborne Irritants

Despite its layered protections, the skin barrier can be compromised by airborne irritants, including those in agricultural environments. Current literature describes several mechanisms by which these irritants disrupt epithelial barrier integrity, which can be grouped into three broad categories: proteolytic degradation of barrier proteins, oxidative stress, and immune activation.

Enzyme allergens such as mite allergens can cleave occludin, induce proteolysis, and increase epithelial permeability while promoting release of inflammatory cytokines such as IL-6 and IL-8 [35]. A unifying feature of many agricultural irritants is their ability to physically and chemically disrupt the skin barrier. Plants such as Ambrosia artemisiifolia (ragweed), Betula spp. (birch), Poa pratensis (Kentucky bluegrass), and Lolium perenne (ryegrass) can damage tight junctions, while diesel exhaust increases reactive oxygen species in the epithelium and causes loss of filaggrin and cytokeratin [35]. Increased barrier permeability allows further irritant penetration, exacerbating damage. Because agricultural workers often experience chronic, repetitive exposure, these effects may accumulate and further compromise barrier integrity.

**Figure 2:**
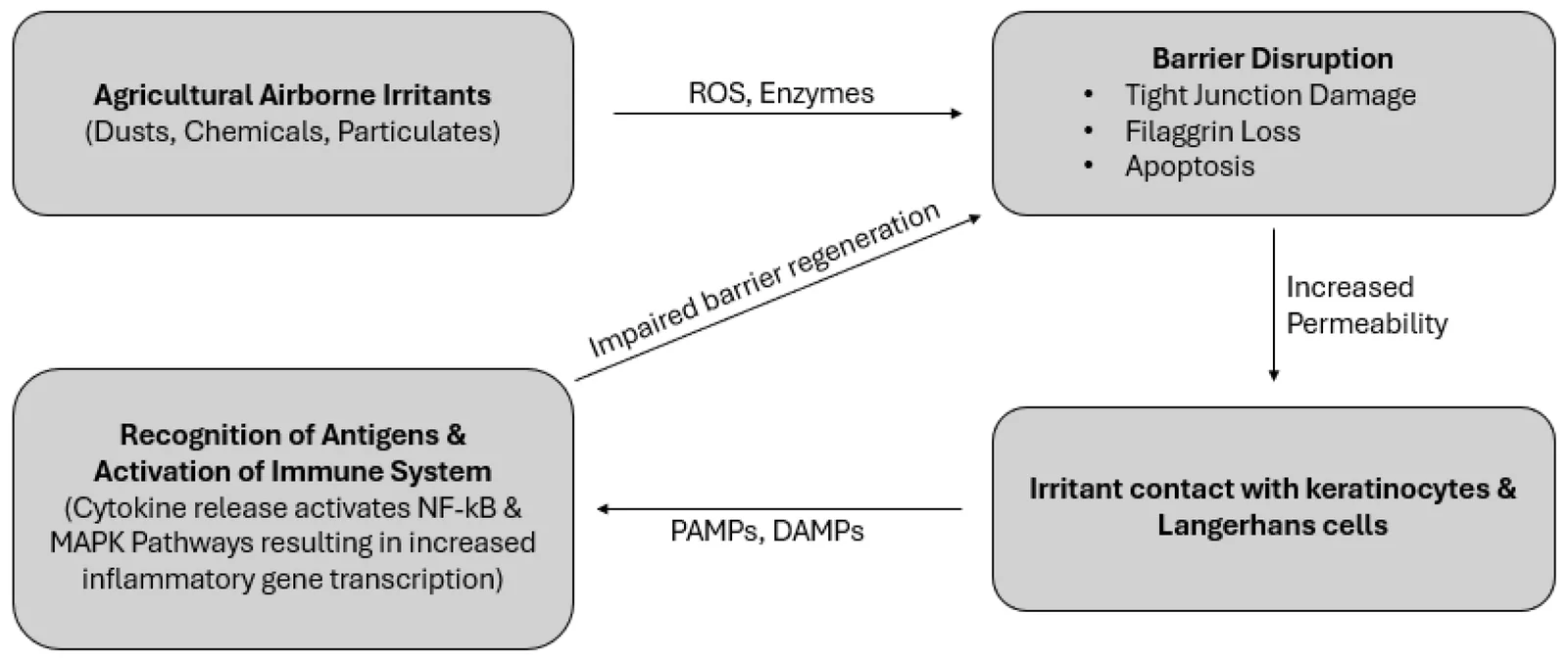
Diagram illustrating proposed mechanisms by which agricultural irritants may disrupt the skin barrier and trigger immune activation. Airborne irritants such as dust, chemicals, and particulates penetrate the epidermis through proteolytic activity and oxidative stress [34,35], damaging tight junctions, reducing filaggrin expression, and inducing apoptosis [19,23]. Barrier disruption may increase epidermal permeability, allowing additional irritants to penetrate and interact with keratinocytes and Langerhans cells. Recognition of pathogen-associated molecular patterns (PAMPs) and damage-associated molecular patterns (DAMPs) by keratinocytes activates NF-κB and MAPK inflammatory pathways, leading to cytokine release. This inflammatory cascade further impairs barrier regeneration [16], perpetuating barrier dysfunction and inflammation. These pathways represent proposed mechanisms and should not be interpreted as establishing a causal relationship between agricultural exposures and cystic acne.

Pesticides represent another group of agricultural irritants with documented effects on the skin barrier. They can impair barrier integrity by reducing tight junction protein expression, altering antioxidant enzyme activity, increasing lipid peroxidation, and inducing apoptosis through reactive oxygen species [16]. Beyond direct barrier damage, pesticide exposure has also been linked to immune-mediated inflammatory disorders [16]. Collectively, these irritants disrupt barrier function through structural breakdown, oxidative stress, and immune activation, highlighting how agricultural airborne irritants may contribute to inflammatory processes implicated in dermatologic conditions, including cystic acne. See Figure 2 for more details regarding mechanisms by which agricultural irritants may disrupt the skin barrier and trigger immune activation.

## 4. Pathophysiology of Cystic Acne

### 4.1. Overview of Acne Development: Sebaceous Gland Activity and Follicular Occlusion

Sebaceous gland activity is largely hormonally controlled, with androgens stimulating sebum production during puberty and adulthood. Beyond volume, sebum composition is increasingly recognized as a key factor in acne development. Dysregulated lipid metabolism, including altered sphingolipid balance, promotes follicular occlusion and pro-inflammatory signaling [36]. Abnormal keratinocyte differentiation also contributes to follicular plugging, creating a microenvironment conducive to acne lesion formation [37]. Increased lipid peroxidation products in sebum promote oxidative stress and enhance local cytokine production, amplifying inflammation [38]. These findings suggest that sebum is not merely a passive byproduct but an active participant in acne pathogenesis. Both the quality and quantity of sebaceous output influence acne susceptibility, providing a rationale for therapies targeting lipid regulation rather than simply suppressing sebum production.

### 4.2. Cutibacterium Acnes Colonization

Once sebaceous and follicular changes occur, *Cutibacterium acnes* colonization becomes a key contributor to acne progression. Although this bacterium is a normal skin commensal, it acquires pathogenic potential within the occluded follicle, where it produces lipases, porphyrins, and short-chain fatty acids that stimulate inflammation [16]. Barrier dysfunction may further facilitate colonization by allowing bacterial proliferation and deeper penetration of microbial products [39]. *Cutibacterium acnes* activates Toll-like receptor 2 (TLR2) and the NLRP3 inflammasome in sebocytes and keratinocytes, increasing IL-1β and IL-8 secretion and sustaining neutrophilic infiltration [40]. Strain variability also contributes, with certain lineages more strongly associated with inflammatory lesions, suggesting that acne severity may depend on microbial composition as well as host response. Importantly, colonization alone does not explain acne, as many individuals harbor *Cutibacterium acnes* without developing disease. This underscores the potential role of environmental and systemic cofactors in shifting the balance from commensalism to pathogenicity.

### 4.3. Role of Environmental Triggers: Irritants Disrupting Skin Barrier Function and Inflammatory Pathways

Environmental exposures, including air pollution, cosmetics, and climate, significantly affect skin health and acne pathogenesis. Pollutants such as particulate matter (PM2.5), sulfur dioxide, and nitrogen oxides disrupt the epidermal barrier by altering lipid composition and impairing keratinocyte function [24]. Deng et al. (2024) further describe how cosmetic occlusion and occupational irritants similarly weaken the barrier and predispose individuals to follicular occlusion [39]. Climatic factors such as humidity and heat contribute by causing keratinocyte swelling, narrowing follicular openings, and increasing sweat-sebum mixing on the skin surface [32]. These disruptions lower the threshold for microbial colonization and exacerbate intrinsic vulnerabilities. Thus, acne is not merely a genetic or hormonal disease but is also shaped by everyday environmental exposures.

**Figure 3:**
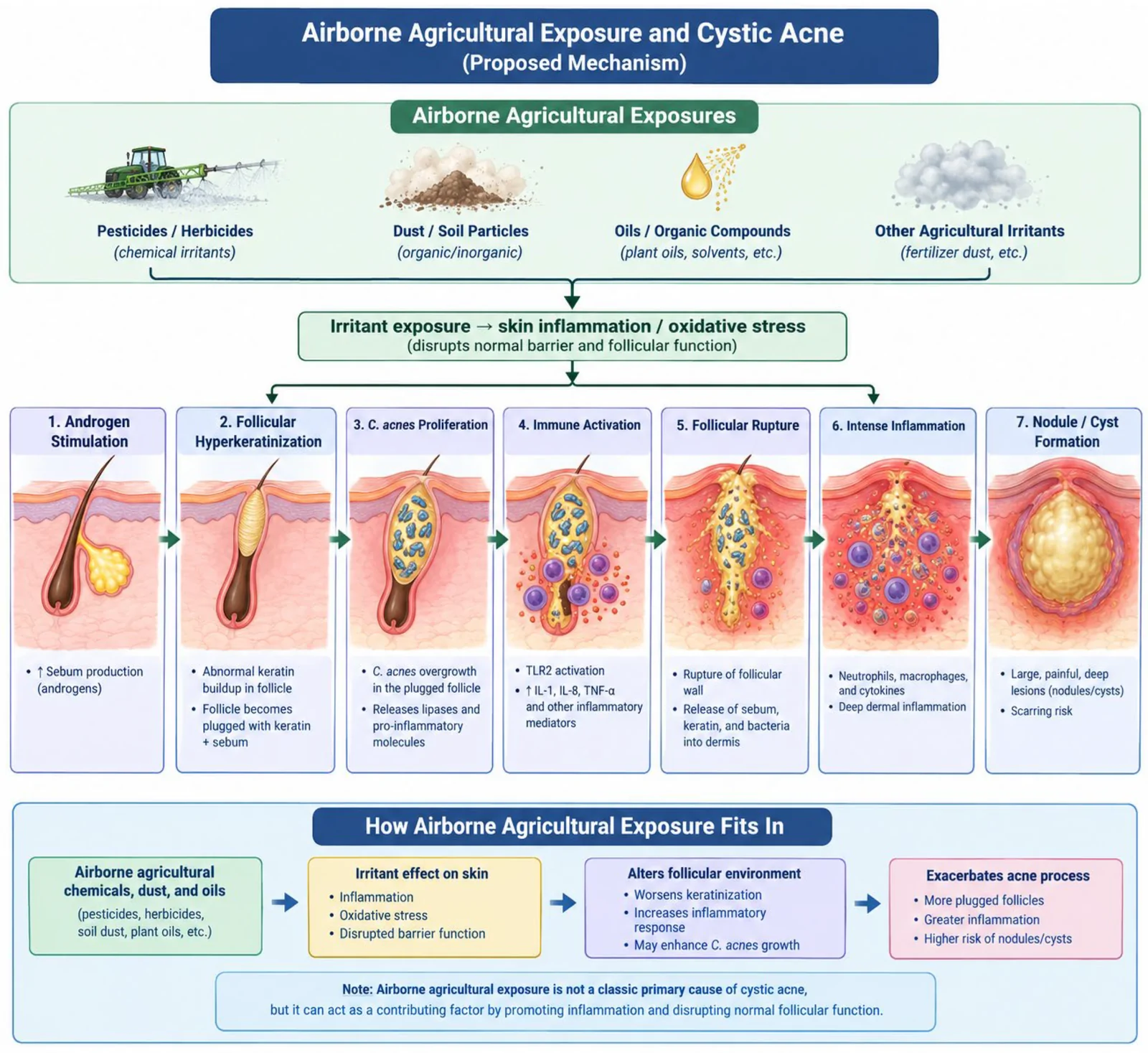
Airborne Pollutant–Induced Inflammatory Pathway in Cystic Acne: Schematic illustrating how airborne agricultural chemicals, dust, and oils can act as cutaneous irritants, causing oxidative stress and disruption of the skin barrier, which promotes local inflammation. This inflammatory environment can increase follicular keratinization and sebum retention, causing the pilosebaceous unit to become obstructed and creating an anaerobic environment that favors *Cutibacterium acnes* proliferation. *Cutibacterium acnes* activates TLR2 on innate immune cells and keratinocytes, stimulating release of pro-inflammatory cytokines such as IL-1, IL-8, and TNF-α, which intensify inflammation around the follicle. Continued inflammation can cause follicular rupture, releasing keratin, sebum, and bacterial products into the dermis and producing the deep, intense inflammatory response characteristic of cystic acne. The arrows demonstrate the progression from agriculture exposures to skin inflammation, follicular changes, and subsequent cyst formation. Note: This figure was AI-generated using OpenAI’s DALL-E model and subsequently curated for scientific accuracy.

Barrier disruption is closely tied to inflammatory responses and oxidative stress, both of which can increase acne severity. Reactive oxygen species that form due to oxidative stress will overwhelm local antioxidant defenses, which results in lipid peroxidation which drives DNA damage in keratinocytes. Oxidative stress also upregulates proinflammatory cytokines such as IL-1, IL-6, and TNF-α, further recruiting immune cells into the follicular environment [40]. Epidemiological studies support these mechanisms, showing a higher prevalence of inflammatory acne in populations exposed to elevated ambient pollution [29]. These findings suggest that inflammation is not simply an endpoint of acne but an amplifier that shapes lesion type and severity. Sustained environmental exposures may therefore predispose individuals to more severe phenotypes, including cystic acne, through direct follicular effects and indirect systemic immune priming (Figure 3).

### 4.4. Cofactor Direct and Indirect Effect on Cystic Acne: Direct Follicular Irritation and Blockage

Direct follicular irritation from cofactors provides an additional pathway to severe acne. Substances such as particulate matter, comedogenic cosmetic ingredients, and mechanical stressors like tight clothing or mask use can obstruct follicular openings [24]. Unlike intrinsic keratinocyte overgrowth, these external agents bypass sebaceous physiology and accelerate occlusion. Climatic factors, particularly humidity and temperature, similarly exacerbate follicular swelling and occlusion [32]. Chronic exposure to airborne pollutants induces oxidative stress and lipid peroxidation within pilosebaceous units, aggravating follicular obstruction and enhancing inflammatory cytokine expression in in vitro skin models [24]. Local cofactors may therefore act synergistically with sebaceous and microbial changes to intensify lesion severity. This may explain why some individuals develop cystic acne under specific environmental or occupational conditions despite similar intrinsic risk factors.

### 4.5. Systemic Immune Priming via Inhalation and Dermal Absorption

Beyond local irritation, systemic effects of cofactors may contribute to cystic acne pathogenesis. Inhaled pollutants induce systemic low-grade inflammation, increasing circulating cytokines that sensitize cutaneous immune responses [25]. Dermal chemical absorption and dietary influences may also contribute through the diet–gut–skin axis, whereby systemic metabolic and immune alterations exacerbate cutaneous inflammation [32]. Recent work suggests that chronic environmental exposures may create a “primed” immune state that lowers the threshold for nodulocystic lesions [39]. This perspective shifts acne research from a purely local skin disorder toward one influenced by systemic physiology and environmental factors and highlights a gap in the literature, as most therapeutic strategies do not address these systemic and environmental contributors.

## 5. Epidemiological Evidence

### 5.1. Rural vs. Urban Acne Prevalence

Findings on rural versus urban acne prevalence are mixed, with confounders such as diet, sun exposure, and socioeconomic status complicating direct comparisons. A cross-sectional study in Thailand found similar rates of moderate-to-severe acne among rural (32.7%) and urban (32.6%) adolescents, with no significant association between dietary habits, particularly milk consumption, and acne severity [41]. In contrast, Dreno et al. found higher acne prevalence in urban areas with higher socioeconomic status than in rural areas [42], while Haider et al. found that patients living in urban areas were 43% more likely to be referred to a dermatologist [43]. Greater access to dermatologic care may therefore contribute to higher reported urban prevalence.

Studies examining diet have found positive associations between acne and intake of fat, sugar, and fast food [44]. Findings on the relationship between sun exposure and acne are mixed, with some studies reporting no association and others reporting increased acne frequency with greater sun exposure [42,45]. Overall, the relationship is multifactorial, and targeted studies are needed to clarify the roles of these confounders.

### 5.2. Occupational Dermatology Factors

There are limited reports on acne and other inflammatory skin disorders among agricultural workers. Krejci et al. reported acne or folliculitis in 24.1% of farmworkers and contact dermatitis in 5.6% [46]. Similarly, a North Carolina study found inflammatory skin disease in 57.2% of farmworkers during the agricultural season, with acne and folliculitis most common (47.7%), followed by contact dermatitis (12.2%). Inflammatory disease diagnoses decreased with age and increased with pesticide exposure [47].

Agricultural and industrial exposures share similarities, particularly regarding contact dermatitis from frequent irritant exposure. Chu et al. reviewed common occupational allergens and the prevalence of occupational contact dermatitis, identifying metals (nickel, cobalt, and chromium), pesticide ingredients (malathion, propiconazole, mercaptobenzothiazole, and formaldehyde), and thiuram mix, a rubber additive, as common allergens among agricultural workers. In contrast, production workers are commonly exposed to adhesives (epoxy resin and colophony), rubber accelerators (thiuram mix and carba mix), metals (nickel and cobalt), and formaldehyde [48].

### 5.3. Environmental Health and Pollution Studies

Several studies have linked airborne pollutants, including PM2.5, PM10, NO_2_, VOCs, and industrial emissions, to increased acne prevalence and severity. A time-series analysis in Xi’an, China, found that short-term increases in PM10, SO_2_, and NO_2_ were associated with increased outpatient visits for post-adolescent acne, with females more sensitive to PM10 than males [27]. Similarly, a Lebanese study found that high NO_2_ exposure and employment near a power plant emitting CO_2_, CO, NO_2_, and PM were associated with higher acne prevalence [25].

While several studies link pollutants to increased acne prevalence and severity, some pollutants, including SO_2_ and ozone (O_3_), may have protective effects. A La Roche-Posay study in Beijing found that increased O_3_ concentrations were associated with lower sebum rates and fewer acne lesions. Similarly, this study and an epidemiological study in Beijing found that higher SO_2_ concentrations were associated with lower sebum rates, fewer lesions, and fewer outpatient visits [27].

Although no direct epidemiological association has been established between agricultural airborne irritants and acne, indirect evidence suggests plausible mechanisms. VOCs are skin irritants that may contribute to barrier dysfunction and inflammation [49]. Agricultural dust contains particulate matter that can disrupt the skin barrier and induce inflammation [24]. Industrial chemicals such as pesticides, herbicides, and combustion products can also disrupt endocrine function; endocrine-disrupting chemicals (EDCs) interfere with hormonal pathways involved in acne development [28]. Agricultural dust and chemicals may therefore plausibly promote acne through inflammation, oxidative stress, and hormonal changes, although direct epidemiological evidence is still needed.

## 6. Mechanistic Links

### Skin Microbiome Alterations

**Figure 4:**
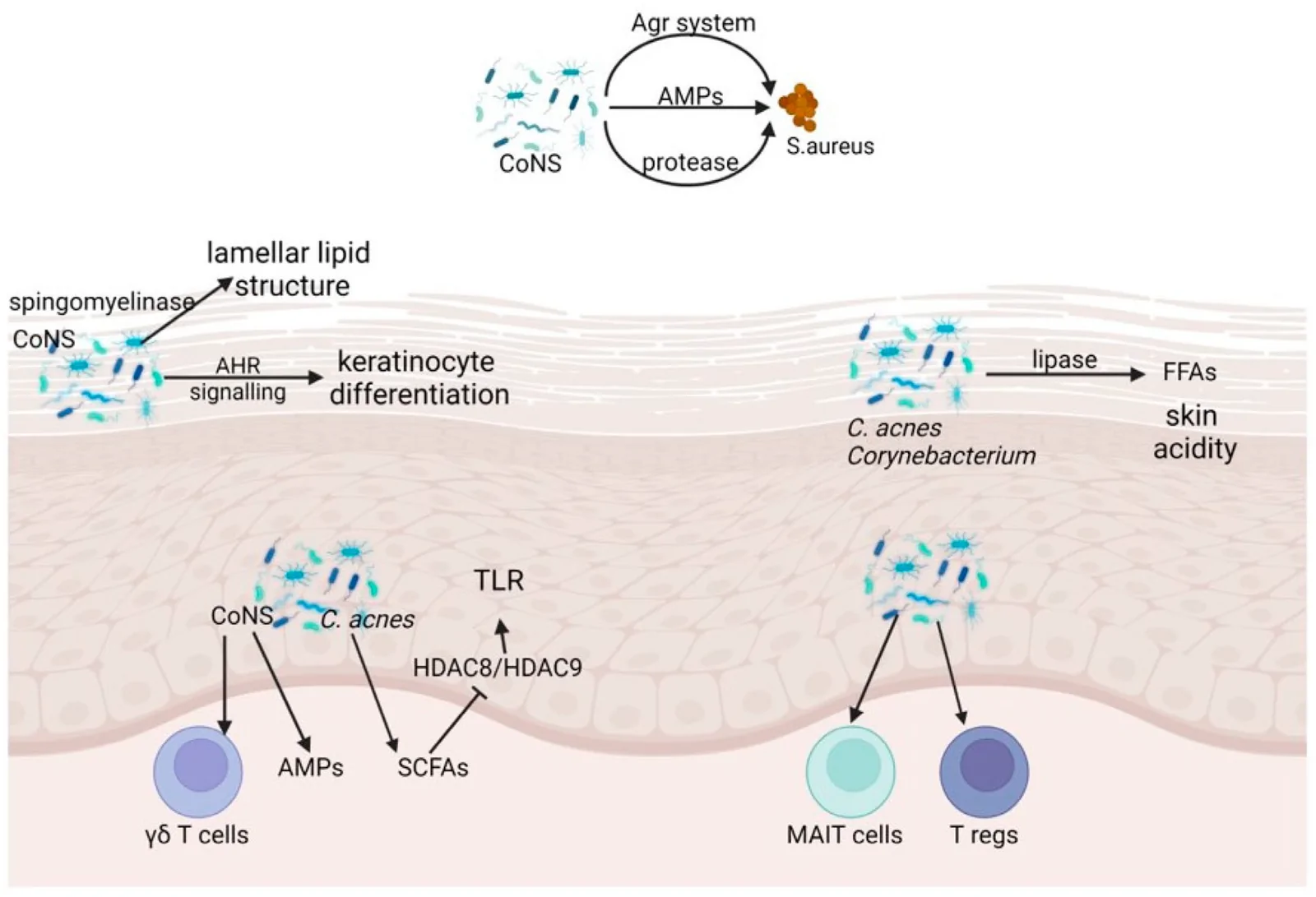
Microbial, physical, chemical, innate, and adaptive immune barrier functions of the skin microbiome. CoNS, coagulase-negative Staphylococcus; Agr, accessory gene regulator; AMPs, antimicrobial peptides; AHR, aryl hydrocarbon receptor; *C. acnes*, *Cutibacterium acnes*; SCFA, short-chain fatty acids; HDAC, histone deacetylases; MAIT, mucosal-associated invariant T cells; T regs, regulatory T cells [52].

Air pollution can disrupt the skin microbiome, which is essential for maintaining barrier function [50,51]. Fine particulate matter (PM2.5, PM10, and smaller particles) often carries lipophilic compounds such as polycyclic aromatic hydrocarbons and volatile organic compounds that penetrate and disrupt the skin’s protective lipid layer [46]. Figure 1 demonstrates how the skin microbiome regulates microbial balance, immune activity, and barrier defense [52]. Continuous exposure to pollutants such as dust and pesticide residues may favor colonization by acne-promoting bacteria [53]. Acne pathogenesis is traditionally attributed to excess sebum production, follicular hyperkeratinization, and *Cutibacterium acnes*. However, increasing evidence emphasizes microbial diversity and strain variability rather than a single pathogenic organism. Environmental pollutants may contribute to dysbiosis by reducing commensal *Cutibacterium acnes* diversity and promoting more virulent ribotypes, particularly RT4 and RT5, which are associated with inflammatory acne lesions [53]. As summarized in Table 3, loss of *Cutibacterium acnes* diversity and dominance of RT4 and RT5 may contribute to acne pathogenesis through biofilm formation, increased virulence and antimicrobial resistance, and immune activation via Toll-like receptor and Th17 pathways [54]. These strains also produce higher levels of virulence factors such as Christie–Atkins–Munch-Petersen (CAMP) factors and porphyrins, generating oxidative stress and local inflammation. Through Toll-like receptor 2 (TLR2) activation on keratinocytes and sebocytes, RT4 and RT5 stimulate release of proinflammatory cytokines including IL-1β, IL-6, and TNF-α, amplifying immune activation [53]. Furthermore, increased lipase activity breaks down sebum triglycerides into free fatty acids that irritate the follicular epithelium and promote hyperkeratinization. Collectively, pollutant-induced barrier disruption and microbial dysbiosis may facilitate dominance of acne-associated *Cutibacterium acnes* ribotypes, linking environmental exposures to inflammation and altered microbial dynamics in acne pathogenesis (Figure 4).

Disruption of the skin barrier is closely linked to reduced filaggrin production, compromising epidermal integrity and facilitating microbial colonization by organisms such as Staphylococcus aureus, which can amplify Th2-skewed inflammation [55,56]. Recent treatments for atopic dermatitis target this pathway, including tapinarof, a topical aryl hydrocarbon receptor (AHR) ligand that activates the AHR/CYP1A1 axis to upregulate filaggrin and involucrin expression [57]. Tapinarof also exhibits antioxidant properties through NRF2 activation and direct reactive oxygen species scavenging, counteracting high-affinity AHR ligands such as particulate matter that promote oxidative stress and cytokine-mediated inflammation [57,58]. Clinical trials demonstrate that systemic absorption of tapinarof remains minimal, even with barrier disruption, and decreases as epidermal function improves. Evidence also supports an association between environmental pollution and inflammatory acne, with higher rates of pollution-associated flares reported in urban populations [59]. Comparative studies have found a higher prevalence of inflammatory than comedonal acne among Asian women, particularly in regions with elevated air pollution [57]. Studies in heavily polluted cities such as Shanghai and Mexico City also found reduced vitamin E and squalene levels among acne patients, suggesting sebum oxidation as a marker of pollution-induced skin damage [60,61]. Because increased sebum oxidation and production are early events in acne pathogenesis, these findings suggest that air pollution exacerbates oxidative stress and barrier dysfunction, contributing to inflammatory acne development and persistence [62].

Endocrine-disrupting chemicals common in agricultural settings have been increasingly implicated in hormonal pathways relevant to acne pathogenesis [28]. Acne vulgaris is a chronic inflammatory disorder of the pilosebaceous unit influenced by hormones including androgens, insulin-like growth factor 1 (IGF-1), estrogens, and corticosteroids [28]. These hormones regulate sebaceous gland differentiation, lipid synthesis, and follicular keratinization, which can be disrupted by exogenous chemical exposures. Endocrine disruptors primarily act by altering endogenous hormone concentrations or interfering with receptor-mediated signaling [29]. Compounds such as bisphenol A and mono-2-ethylhexyl phthalate act as androgen receptor agonists, enhancing lipogenic gene expression and cholesterol synthesis, increasing sebum production and predisposing to follicular occlusion. In contrast, phytoestrogenic endocrine disruptors such as resveratrol and genistein may have protective effects by attenuating peroxisome proliferator-activated receptor gamma (PPARγ) activity and restoring estrogen-regulated transforming growth factor beta expression, counteracting androgen-induced sebocyte maturation [30]. These mechanisms suggest that chronic exposure to agricultural endocrine disruptors may contribute to hormonal imbalance, increased sebaceous activity, and local inflammation, ultimately promoting acne development and persistence.

## 7. Public Health and Clinical Implications

### 7.1. Occupational Health Considerations

Agricultural workers face substantial occupational risk from dermal exposure to pesticides, organic dust, and bioaerosols, which can cause inflammatory skin conditions and systemic toxicity. Accurate risk assessment requires quantifying potential dermal exposure (PDE) and actual dermal exposure (ADE) using methods such as patch dosimetry and hand-washing studies and calculating a Margin of Safety (MOS) against the Acceptable Operator Exposure Level (AOEL) to identify unsafe scenarios (MOS < 1) [17]. Real-world exposure durations should also be considered, as default 8 h assumptions may overestimate absorbed doses by up to 80% and correlate less accurately with biomarkers such as urinary ETU [63]. International guidelines (OECD, EFSA, EPA) recommend tiered or “Triple Pack” testing to improve dermal absorption estimates beyond the default 10% factor used in many jurisdictions [64].

Critical gaps in occupational safety include low PPE compliance, inadequate protective equipment, and insufficient hygiene infrastructure, all of which are associated with higher rates of rashes and dermatitis [65,66]. Glove use ranges from 15% to 55% in the United States, with less than 5% of vineyard workers reporting consistent use, while only about one-third of Indian farmers use protective clothing [17,63,67]. Thermal discomfort and limited availability are common barriers. Unsafe practices, including mixing pesticides with bare hands, checking nozzle speed while machinery is running, and clearing blockages by mouth, further increase exposure [17]. Delayed decontamination after work may also prolong skin contact with irritants.

Hygiene deficits are similarly prevalent; approximately one-fifth of farmworkers lack hand-washing facilities, while soap and paper towels are often unavailable [66]. These conditions are associated with increased severe rash prevalence. Regular dermatologic screening should therefore be incorporated into farmworker health programs, as current screening tools such as skin-rash charts are underused. One study found that 37% of workers reported a rash, with pregnant workers experiencing a 4.7-fold higher risk of extensive rash [66]. Collectively, inadequate PPE, poor hygiene, limited exposure assessment, and insufficient dermatologic surveillance represent substantial gaps in occupational protection for farmworkers exposed to pesticide-laden dust, organic particulates, and bioaerosols.

### 7.2. Preventive Strategies

Preventive strategies for farmworkers should combine consistent PPE use, farm-hygiene practices, and potentially topical barrier or antioxidant preparations to strengthen the skin’s defenses. Gloves, long-sleeved coveralls, and boots can reduce dermal pesticide exposure by 27–65%, depending on garment type, yet compliance remains poor, with only 5–15% of workers regularly using gloves during mixing and application and fewer than half reporting protective clothing [67]. Farm-level hygiene measures, including accessible hand-washing stations, immediate post-exposure washing, and changing out of work clothes within 30 min of leaving the field, are associated with a three-fold reduction in severe rash risk. Hand-washing alone accounts for much of the exposure reduction and decreased body-rash prevalence observed in field studies [66].

Barrier creams are applied before work as part of a three-step occupational skin-protection program and are intended to form a protective film that limits hazardous substance penetration. However, Kütting et al. (2003) found a lack of placebo-controlled randomized trials under real workplace conditions and reported conflicting results, with many studies showing no significant benefit over vehicles or even increased chemical penetration [68]. The authors concluded that evidence remains insufficient to establish a preventive effect of barrier creams in occupational settings and called for well-designed field trials evaluating product type, application frequency, and education before recommending them as reliable preventive measures [68].

## 8. Discussion

A major weakness of the existing evidence is its reliance on cross-sectional surveys and brief field trials, which limit assessment of incidence, temporal trends, and causal links between pesticide exposure, skin barrier disruption, and health outcomes, particularly in susceptible populations such as pregnant farm workers [66]. Future research should use prospective, longitudinal designs that follow rural cohorts across multiple growing seasons, quantify exposure through dermal dose and biomarkers, and systematically assess dermatologic outcomes using validated tools such as the skin-rash diagram [66].

An interdisciplinary “One Health” approach could integrate dermatologic phenotyping by dermatologists, exposure assessment by occupational physicians, and evaluation of pesticide practices, formulations, and crop-specific risks by agronomists. Intervention studies should also evaluate workplace controls, including improved hand-washing facilities, training programs, and engineering controls, based on both exposure reduction and clinical outcomes [66]. Well-characterized rural cohorts integrating dermatologic assessments, exposure measurements, and farming practices could provide longitudinal data to inform regulatory policy, guide prevention, and reduce occupational skin disease among farm workers (Table 4).

## 9. Conclusions

Cystic acne has traditionally been understood as a multifactorial condition driven by hormones, genetics, and microbial imbalance. However, this review highlights that environmental factors, particularly airborne agricultural irritants, may play a greater role than previously recognized. Continuous exposure to organic dusts, microbial endotoxins, pesticides, volatile compounds, and animal-derived particulates can damage the skin barrier, increase oxidative stress, and activate inflammatory pathways that may intensify acne severity. These exposures may also alter the skin microbiome and promote systemic inflammation through inhalation and dermal absorption. Together, these findings suggest that cystic acne reflects the broader interaction between the skin, environment, and immune system.

Despite emerging evidence, research directly connecting agricultural exposures to cystic acne remains limited. Existing studies often rely on small samples and do not account for confounding factors such as diet, sun exposure, or access to dermatologic care. Larger, targeted studies are needed to identify specific airborne agents that contribute to barrier disruption and inflammation. Laboratory models could also clarify how chronic exposure affects lipid oxidation, cytokine production, and microbiome composition within the pilosebaceous unit.

Agricultural workers and rural populations may benefit from greater awareness of skin-protection strategies, including barrier-supportive skincare, PPE, and post-exposure cleansing. Recognizing cystic acne as a potential occupational health concern may also inform public health policies aimed at reducing harmful exposures. Continued collaboration among dermatology, environmental health, and agricultural sciences will be essential to understanding and preventing environmental contributors to cystic acne.

## Figures and Tables

**Table 2:** Summary of agricultural and environmental pollutants, their sources, primary exposure routes, and reported cutaneous effects.

Pollutant/Irritant	Main Sources	Primary Exposure Route(s)	Reported Skin Effects	Refs
Organic dusts (grain, hay, soil dust)	Grain handling, harvesting, hay storage, soil disturbance	Dermal contact; inhalation	Barrier penetration by plant particles, heavy metals, and PAHs; mechanical abrasion; dermatitis and inflammation	[11,13]
Mold spores (e.g., *Aspergillus fumigatus*)	Stored hay and grain, damp organic material	Inhalation	Th17/Th2 activation, increased inflammatory cytokines, impaired regulatory T-cell function	[12]
Microbial endotoxins (LPS)	Gram-negative bacteria in organic dust and bioaerosols, livestock environments	Inhalation; dermal contact	Systemic immune activation; direct activation of skin immune cells; skin irritation and worsening of existing dermatoses	[6,14]
Pesticides (e.g., organophosphates, pyrethroids)	Mixing, loading, and spraying; spray drift; residues on crops and dust	Dermal contact (primary); inhalation	TLR activation and alarmin release; increased IL-6, IL-1β, and TNF-α; reduced tight junction proteins; oxidative stress; dermatitis	[6,15,16,17]
Herbicides (glyphosate, glufosinate with surfactants)	Herbicide application	Dermal contact	Chemical burns, bullae, and necrosis; greater penetration through damaged or aged skin	[18]
Fertilizer- and manure-related irritants (e.g., ammonia)	Farmyard manure, cow dung, livestock housing	Dermal contact; airborne irritant exposure	Irritation, allergic reactions, chemical burns; higher skin disease prevalence without protective equipment	[19]
Volatile organic compounds (e.g., toluene)	Barns and storage facilities	Inhalation; dermal contact	Filaggrin downregulation, increased TEWL, ROS generation, squalene oxidation, AhR activation	[20,21,22]
Heavy metals (e.g., lead, cadmium)	Grain dust; co-exposure with VOCs	Dermal contact; inhalation	Suppressed antioxidant defenses and amplified oxidative injury	[11,22]
Animal-derived particulates (dander, dried manure, feather fragments)	Livestock and poultry housing	Dermal contact; inhalation	Allergen penetration of the barrier; dendritic cell and macrophage activation; inflammasome activation	[19,23]
Particulate matter (PM2.5, PM10)	Agricultural dust, combustion, industrial emissions	Dermal deposition (including follicular); inhalation	Altered barrier lipids, follicular occlusion, oxidative stress; systemic low-grade inflammation	[24,25,26]
Gaseous pollutants (NO_2_, SO_2_, O_3_)	Combustion, power plants, industrial emissions	Inhalation; skin surface contact	NO_2_ associated with increased acne; SO_2_ and O_3_ associated with lower sebum and fewer lesions in some studies	[25,27]
Endocrine-disrupting chemicals (e.g., BPA, phthalates, pesticide-derived EDCs)	Pesticides, herbicides, combustion products	Dermal contact; inhalation; ingestion	Altered hormone levels and receptor signaling; androgen receptor agonism with increased sebum production	[28,29,30]

PAHs, polycyclic aromatic hydrocarbons; LPS, lipopolysaccharide; TLR, Toll-like receptor; TEWL, transepidermal water loss; ROS, reactive oxygen species; AhR, aryl hydrocarbon receptor; VOCs, volatile organic compounds; EDCs, endocrine-disrupting chemicals; BPA, bisphenol A.

**Table 3:** Key factors in acne development and progression [55].

Factors Contributing to Acne	Description
Microbiome alternations	Loss of *Cutibacterium acnes* diversity; dominance of pathogenic strains (RT4, RT5) causing biofilm formation; increased virulence and resistance.
Immune system activation	Immune responses triggered (Toll-like receptors, Th17 pathway) leading to increased IL-1 and IL-17 cytokines.
Follicle changes	Genetic predisposition to hyperseborrhea, hyperkeratinization, and androgen sensitivity.
Environmental exposures	External factors like diet, stress, and pollutants (exposome) that exacerbate acne symptoms.

**Table 4:** Summary of preventive strategies for occupational skin protection [17,63,64,65,66,67,68].

Prevention Measure	Description	Evidence	Compliance/Feasibility	Key Findings/Observations
Barrier/Skin-Protection Creams (Pre-Work)	Pre-exposure film applied to skin before tasks.	Mixed: some decreased sensitization; others show no benefit or increased solvent penetration.	Low routine use; real-world controls are often absent.	Chelating-agent creams reduce metal allergy; vehicles can perform similarly to active formulations; overall field benefit unproven.
Skincare Educational Programs	Training on hand hygiene, moisturizers, and barrier-cream use.	Moderate: Decreased hand-disinfectant use; Decreased transepidermal water loss (TEWL).	Variable; depends on employer support and reinforcement.	Structured programs in wet-work settings show benefit; impact of individual elements is difficult to isolate in evaluations.
Personal Protective Equipment (PPE)	Chem-resistant gloves, coveralls, boots, goggles for mixing/spraying/handling.	Strong: ≈27% decreased dermal dose (gloves) to ≈65% (gloves + coveralls); >90% decreased hand contamination.	Poor adherence (<30% for full ensembles) due to heat, discomfort, and supply constraints.	Waterproof fabrics outperform cotton; training increases PPE uptake and correct donning/doffing, further lowering contamination.
Hand-Washing/Hygiene	Soap hand-washing after handling and before breaks; access to wash stations.	High: repeatedly identified as the single most effective intervention for decreased absorbed dose and rash risk.	Moderate: stations and supplies frequently lacking.	With realistic exposure durations, absorbed dose falls by ~80%; access to stations reduces rash odds ~1.76× compared with no access.
Combined PPE & Hygiene	Full PPE with regular hand-washing; post-work showering.	Very high: near-zero actual dermal exposure (ADE) and negligible penetration factors for many body regions.	Better than PPE alone when facilities and training are provided; still limited by compliance barriers.	ADE values ~10–13 µg drop to ≤0.6 µg; Margin of Safety (MOS) typically >1 (safe levels).
Administrative Controls	Limit task time, rotate jobs, enforce breaks to reduce cumulative exposure.	Limited skin-outcome evidence; most data from non-agricultural settings.	Rarely applied in field agriculture due to labor and cost constraints.	Recognized control but difficult to implement in small-scale or informal farms; effectiveness depends on managerial oversight.
Engineering/Barrier Controls	Enclosed mixing stations, mechanized applicators, physical barriers to reduce contact.	Limited published field evidence but conceptually effective.	Low feasibility where capital and equipment are scarce.	Effective when implemented; scalability constrained by up-front cost, maintenance, and equipment availability.
Banning/Substituting Hazardous Pesticides	Remove highly toxic active ingredients; substitute safer alternatives.	Highest level of control when enforced; exposure source eliminated.	Variable: enforcement gaps in low-income regions; substitution incomplete.	Bans eliminate risk, but some prohibited chemicals persist in practice; surveillance and enforcement remain necessary.
Pollution-Aware Skincare	Barrier/antioxidant moisturizers & cleansers against pollutants.	Emerging evidence; early studies suggest benefit.	Not yet standard in occupational programs.	Promising adjunct in high-pollution environments; supports worker education and clinician awareness about pollutant exposure.
Training/Safety Education	Formal instruction on hazards, PPE, hygiene, risk perception.	Moderate–high: improves PPE compliance; reduces unsafe behaviors.	Variable: frequency, language, cultural relevance influence impact.	Linked to better PPE use and safer practices; PPE access is often lower for minority or migrant workers.

## Data Availability

No new data were created or analyzed in this study. Data sharing is not applicable to this article.

## Abbreviations

The following abbreviations are used in this manuscript:

NF-κB	Nuclear factor kappa B
VOCs	Volatile organic compounds
PAMPs	Pathogen-associated molecular patterns
DAMPs	Damage-associated molecular patterns
ROS	Reactive oxygen species
EDCs	Endocrine-disrupting chemicals
CAMP	Christie–Atkins–Munch-Petersen
IGF-1	Insulin-like growth factor 1
PPARγ	Peroxisome proliferator-activated receptor gamma
PDE	Potential dermal exposure
ADE	Actual dermal exposure
